# Comparing the Effect of Subcutaneous and Intradermal Mesotherapy on Pain and Function in Knee Osteoarthritis: A Randomized Clinical Trial: Short title: Subcutaneous vs. Intradermal Mesotherapy in Knee Osteoarthritis

**DOI:** 10.31661/gmj.v15i.4095

**Published:** 2026-07-12

**Authors:** Parisa Taheri, Soroush Sadri, Maryam Behroozinia1

**Affiliations:** ^1^ Department of Physical Medicine and Rehabilitation, School of Medicine, Isfahan University of Medical Sciences, Isfahan, Iran

**Keywords:** Osteoarthritis, Knee, Randomized Clinical Trial, Mesotherapy, Pain

## Abstract

**Background:**

Knee osteoarthritis (KOA) is the most common form of osteoarthritis and is associated with pain and functional impairment. Mesotherapy, which involves localized intradermal or subcutaneous injection of active agents, may provide targeted pain relief. This study aimed to compare the effects of intradermal and subcutaneous mesotherapy on pain and function in patients with KOA.

**Materials and Methods:**

A single-center, randomized, assessor-blinded clinical trial was conducted in Isfahan, Iran, between 2023 and 2024 among patients with mild-to-moderate KOA. Each patient received six sessions of intradermal or subcutaneous piroxicam-lidocaine mesotherapy at six acupuncture points over three weeks (two sessions per week). Pain and function were assessed at baseline, immediately after treatment, and one month after treatment. Statistical analyses included chi-square tests for qualitative variables, independent-samples t-tests for quantitative variables, and repeated-measures analysis of variance to assess changes over time. Statistical significance was set at P<0.05.

**Results:**

A total of 50 patients were included. The demographic characteristics and baseline pain and function scores did not differ significantly between the two groups. At the one-month follow-up, the intradermal group showed significantly greater improvement, with a mean difference in pain score reduction of 1.6 points (95% CI: 0.57 to 2.67, P=0.003) compared to the subcutaneous group. For dysfunction scores, the intradermal group demonstrated immediate post-treatment benefits (mean difference: 9.4 points, 95% CI: 1.36 to 17.44, P=0.02), with sustained improvement at the one-month post-treatment follow-up (mean difference: 15.5 points, 95% CI: 7.95 to 23.01, P<0.001). Repeated measures ANOVA showed that pain and dysfunction scores significantly changed over time in both groups (P<0.001).

**Conclusion:**

Both subcutaneous and intradermal mesotherapy appeared to be effective short-term treatments for patients with KOA. However, intradermal mesotherapy demonstrated greater effectiveness at the one-month follow-up, with superior pain reduction and functional improvement compared with subcutaneous mesotherapy.

## Introduction

The knee is the most commonly symptomatic joint affected by osteoarthritis [1]. The prevalence of knee osteoarthritis (KOA) among individuals aged 40 years and older is estimated to be approximately 22.9%, with a higher rate observed in women compared to men [2]. Its incidence and overall burden are rising due to aging populations, increased life expectancy, and the growing prevalence of obesity [3].

KOA is characterized by the progressive degeneration of articular cartilage, inflammation of the synovial membrane, and the formation of osteophytes [4]. Clinically, it presents with joint pain, fatigue, movement difficulties, swelling, and tenderness [5]. Management of KOA includes both non-surgical and surgical approaches. Conservative treatments encompass pharmacological and non-pharmacological interventions, such as medications, physical therapy, transcutaneous electrical nerve stimulation (TENS), and intra-articular injections, including corticosteroids and hyaluronic acid [6].

Mesotherapy has recently emerged as a potential treatment modality for KOA. This minimally invasive technique involves intra- or subcutaneous injections of a liquid mixture containing pharmaceutical and homeopathic medications, plant extracts, vitamins, and other bioactive compounds. These substances gradually diffuse into the underlying tissues, exerting localized therapeutic effects [7, 8]. The active ingredients interact with the mesoderm, as well as cutaneous nervous and cellular structures, contributing to the drug-sparing effect characteristic of mesotherapy [9]. The procedure is generally well tolerated, with only minor and transient adverse effects such as allergic reactions, pain, and ecchymosis. A key advantage of mesotherapy over systemic treatments is its slow diffusion mechanism, which enables the administration of lower drug doses while maintaining therapeutic efficacy [10].

This approach may be particularly beneficial for patients with comorbidities, especially when nonsteroidal anti-inflammatory drugs (NSAIDs) are contraindicated. Evidence suggests that mesotherapy is more effective in managing acute pain than chronic pain, although its role in chronic KOA remains beneficial, often requiring additional treatments for sustained improvement [8]. In clinical practice, both intradermal and subcutaneous mesotherapy techniques are used; however, their comparative efficacy in KOA management remains unclear. While some studies have reported positive outcomes for both approaches individually, direct comparisons are scarce.

Therefore, the present study aimed to evaluate and compare the effects of intradermal and subcutaneous mesotherapy on pain reduction and functional improvement in patients with mild-to-moderate KOA. This comparison may help clarify whether one technique offers superior therapeutic benefits and inform clinical decision-making in routine practice.

## Materials and Methods

### Study Design

A single-center, randomized, assessor-blinded, controlled trial was conducted at the Physical Medicine and Rehabilitation clinics affiliated with Isfahan University of Medical Sciences. The study enrolled patients who were clinically diagnosed with KOA by a physiatrist between 2023 and 2024. The study protocol received approval from the Ethics Committee of Isfahan University of Medical Sciences (IR.ARI.MUI.REC.1402.163) and was registered on the Iranian Registry of Clinical Trials (IRCT) on October 10, 2023 (registration number: IRCT20231003059603N1).

### Patients

**Figure-1:**
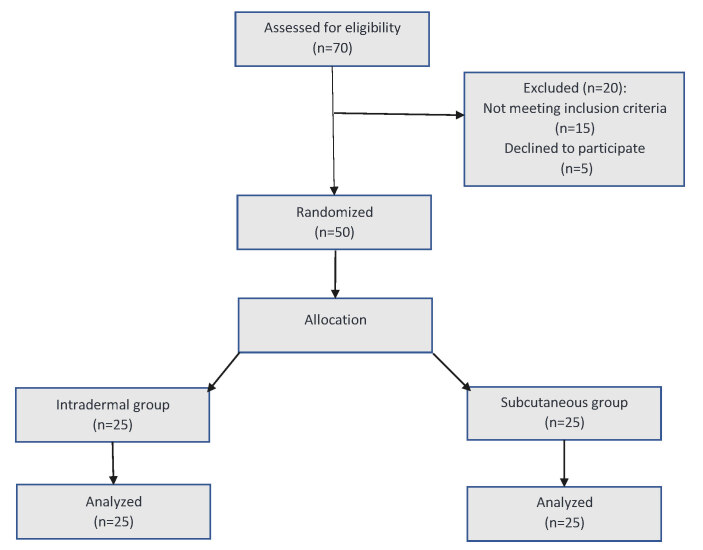
Figure 1. Participant recruitment and retention process

A detailed depiction of the patient recruitment and retention process is presented in Figure-1. A convenience sampling method was used. Patients who visited the Physical Medicine and Rehabilitation clinics with complaints of chronic knee pain were screened for eligibility. Those who met the inclusion criteria were consecutively approached by the research team. An experienced physiatrist made the clinical diagnosis of KOA based on the criteria of the American College of Rheumatology, which include chronic knee pain lasting more than six weeks and at least three of the following: age over 50, crepitus with active motion, tenderness on bony palpation, bony thickening or growth, and absence of local heat on palpation. Inclusion criteria: Patients aged 40–70 years with mild to moderate KOA based on the Kellgren-Lawrence scale and symptoms persisting for at least six weeks. Severe KOA cases were not included, as they are more commonly candidates for surgical management, and conservative injectable treatments such as mesotherapy are primarily indicated for mild to moderate disease. Exclusion criteria: History of knee surgery; receipt of rehabilitation for knee pain within the last three months; presence of neurologic, infectious, malignancy, or cognitive disorders; rheumatologic, nephrological, or cardiovascular diseases; gout, neuropathy, radiculopathy, or nerve injury; BMI greater than 42; and pregnancy. After selecting the patients, a physical medicine and rehabilitation resident informed them about the study’s objectives, methods, existing evidence, potential benefits, and possible complications of the procedure. Participation was limited to those who provided written informed consent. Initially, personal information, including age, sex, BMI, and osteoarthritis grade, was documented. Following this, pain intensity was evaluated using the visual analog scale (VAS), and functional status was assessed with the Western Ontario and McMaster Universities (WOMAC) questionnaire. Sample Size To determine the minimum required sample size, the formula for comparing two independent means was used. A significance level (α) of 0.05, corresponding to a 95% confidence level, and a statistical power (1−β) of 0.90 were considered. Based on the findings of Farpour et al [11]. Based on reported mean ± standard deviation values of 4.74 ± 2.26 and 2.96 ± 1.45 for pain intensity (VAS) in the two groups, respectively, the estimated sample size was 24 participants per group. To account for potential dropout, the final sample size was adjusted to 25 participants per group.

### Randomization and Allocation Concealment

Participants were randomly assigned to one of two intervention groups. A random allocation sequence was generated using Microsoft Excel by creating 50 random numbers and sorting them in ascending order. Based on this sequence, the first 25 participants were assigned to the intradermal mesotherapy group and the remaining 25 to the subcutaneous mesotherapy group. Allocation was performed by an independent researcher not involved in outcome assessment.

### Blinding Procedure

The outcome assessors were unaware of treatment allocation to prevent bias. Blinding was achieved by using coded study IDs, with only the treatment team having access to group assignments. Due to the nature of the intervention, participants and the treating physician were aware of group allocation, while only the outcome assessors were blinded.

### Interventions

In both intervention groups, mesotherapy was performed over 6 sessions, 2 per week (Saturday and Tuesday), over 3 weeks, by the same physician. The cocktail in both groups was also the same, including 20 milligrams of piroxicam (1 mL, Feldene, Razak Pharmaceutical Co., Iran) combined with 2 milliliters of lidocaine 2% (Lidocaine 2%, Darou Pakhsh Pharmaceutical Mfg. Co., Iran). In both groups, after disinfecting the skin while the patient was supine, a sterile, single-use 3 mL syringe with a 27-gauge × 4 mm needle was used for injection. In the intradermal group, the needle was inserted tangentially at a depth of 1–2 mm, just beneath the epidermis, at an angle of approximately 15 degrees to the skin, resulting in a small bleb confirming intradermal injection. In the subcutaneous group, the needle was inserted perpendicularly to the skin at a depth of 4 mm, with no visible bleb formation. At the end of each session, patients were observed for nearly 3 minutes to exclude adverse reactions. The points of injection were the same in both groups, consisting of six acupuncture points: SP9, SP10, ST34, ST35, EX-LE4, and LR8. The locations of these points are as follows:

• SP9: Located below the knee, in the depression at the lower edge of the medial condyle of the tibia, anterior to the bulk of the gastrocnemius muscle.

• SP10: When the knee is flexed, it is found two cun above the superomedial border of the patella, over the protuberance of the medial thigh.

• ST34: Situated on a line connecting the anterosuperior iliac spine to the superolateral border of the patella, two cun above the superolateral border of the patella, in a depression within the vastus lateralis muscle.

• ST35: When the knee is flexed, it is located in the depression lateral to the patellar ligament.

• EX-LE4: With the knee slightly flexed, it is found in the depression below the patella, medial to the patellar ligament.

• LR8: With the knee flexed, it is located in the groove at the medial end of the knee joint crease, between the tendons of the semimembranosus and semitendinosus muscles.

All participants were instructed to modify their lifestyles and perform quadriceps-strengthening and calf-stretching exercises three times a day. Compliance was assessed through phone calls.

### Assessments

Clinical evaluation of pain and functional status was performed using the VAS and WOMAC questionnaires, respectively. The VAS is a scale from 0 to 10, with 0 signifying no pain and 10 indicating the highest level of pain. The patient marks a point on the line that corresponds to their level of pain. Its validity and reliability in pain-related research have been well established [12]. The WOMAC questionnaire consists of 24 items: five questions related to pain, two questions assessing stiffness, and 17 questions related to the patient’s physical function. Each item is rated on a scale from 0 to 4, with lower scores representing fewer symptoms and better functional status. The total WOMAC score is 0 to 96. Eftekhar-Sadat et al. have validated and established the reproducibility of the Persian version of the WOMAC Questionnaire, reporting high internal consistency (Cronbach’s alpha > 0.95) [13]. Scores were documented immediately following treatment and after one month. Data from both time points were gathered, analyzed, and compared.

### Statistical Analysis

Following data collection, data were imported into IBM SPSS Statistics for Windows, version 27.0 (IBM Corp., Armonk, NY, USA) for statistical analysis. Descriptive statistics, including frequency, percentage, mean, and standard deviation, were used. The Kolmogorov-Smirnov test showed that pain and functional disability scores were normally distributed in both groups at all three time points; therefore, parametric tests were used. The chi-square test was used to examine qualitative variables, while independent-samples tests, including Levene’s test for equality of variances and the t-test for equality of means, were used to analyze quantitative variables. To evaluate changes in scores over time between the two groups, a generalized linear model with repeated-measures analysis of variance was applied. When significant effects were found, the LSD (Least Significant Difference) post hoc test was used to compare the time points within each group. Cohen’s d was used, and the Point Estimate and 95% Confidence Interval were reported for effect size. A significance level of 0.05 was adopted for all statistical tests.

## Results

**Figure-2:**
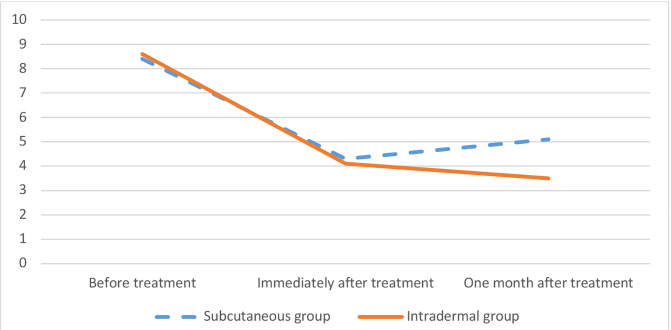
Figure 2. Mean Visual Analog Scale (VAS) pain scores at different time points in the two groups

**Figure-3:**
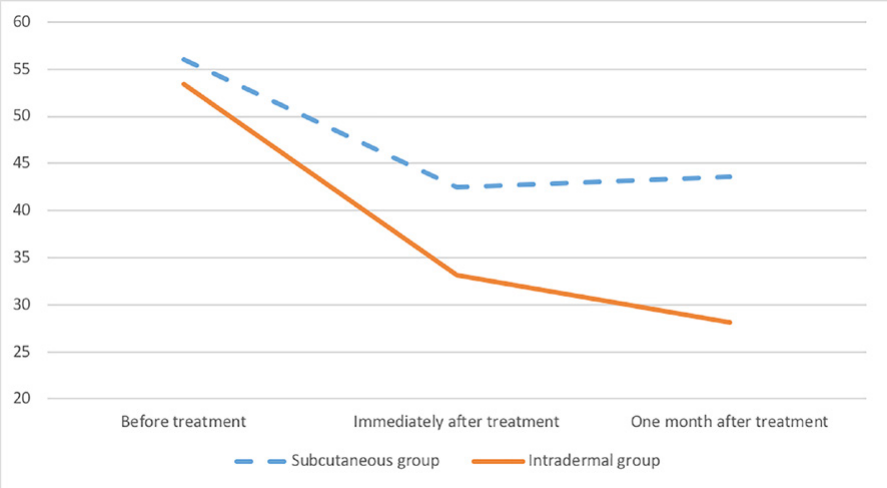
Figure 3. Mean dysfunction scores at different time points in the two groups

**Table:** Table1. Demographic and Baseline Clinical Characteristics of the Two Treatment Groups

Variable	Intradermal group	Subcutaneous group	P-value
Age	60.44 ± 9.22	62.88 ± 7.83	0.32
BMI*	29.88 ± 6.08	27.3 ± 4.66	0.1
Gender†			
Male	11 (44)	10 (40)	0.77
Female	14 (56)	15 (60)	
Severity of OA†			
Mild	10 (40)	12 (48)	0.57
Moderate	15 (60)	13 (52)	

^*^ Mean ± standard deviation (SD); † Number (percentage)

**Table:** Table2. Mean Visual Analog Scale (VAS; 0-10 Pain Scale) Scores at Different Time Points in the Two Groups

Time	Subcutaneous group (n=25) Mean±SD	Intradermal group (n=25) Mean±SD	P-value	Mean difference (CI)	Effect Size (Cohen’s d)
Before Treatment	8.4±1.68	8.6±1.68	0.68	-0.2 (95% CI:-1.16 to 0.86)	-0.119 (95% CI:- 0.673 to -0.437)
Immediately After Treatment	4.28±1.59	4.12±1.69	0.73	-0.2 (95% CI:-0.77 to 1.09)	0.097 (95% CI: -0.458 to 0.625)
One Month After Treatment	5.08±1.73	3.46±1.93	0.003	1.6 (95% CI:0.57to 2.67)	0.885 (95% CI: 0.293 to 1.469)
P-value	<0.001	<0.001		

**CI:** 95% Confidence Interval

**Table:** Table3. Mean Dysfunction Scores Based on the Western Ontario and McMaster Universities Osteoarthritis Index (WOMAC; 0-96) at Different Time Points in the Two Groups

Time	Subcutaneous group (n=25) Mean±SD	Intradermal group (n=25) Mean±SD	P-value	Mean difference (CI)	Effect Size (Cohen’s d)
Before Treatment	56.04±10.8	53.44±16.02	0.5	2.6 (95% CI: -5.17 to 10.37)	0.190 (95% CI: -0.366 to 0.745)
Immediately After Treatment	42.52±15.09	33.12±13.12	0.02	9.4 (95% CI: 1.36 to 17.44)	0.665 (95% CI: 0.092 to 1.232)
One Month After Treatment	43.58±12.21	28.1±12.46	<0.001	15.5 (95% CI: 7.95 to 23.01)	1.256 (95% CI: 0.599 to 1.901)
P-value	<0.001	<0.001			

**CI:** 95% Confidence Interval

A total of 50 patients with KOA were enrolled, with 25 patients in each treatment group. The mean age was 60.4±9.2 years in the intradermal group and 62.9±7.8 years in the subcutaneous group. Most patients in both groups were female, and most had moderate osteoarthritis. The two groups were comparable in terms of age, body mass index (BMI), sex distribution, and osteoarthritis severity, with no statistically significant differences (P>0.05). Demographic and baseline clinical characteristics are summarized in Table1. As shown in Table2, the independent-samples t-test showed that mean pain scores before treatment (P=0.68) and immediately after treatment (P=0.73) did not differ significantly between the two groups. However, one month after treatment, the mean pain score was significantly lower in the intradermal mesotherapy group than in the subcutaneous mesotherapy group (P=0.003). Repeated-measures ANOVA showed that mean VAS scores changed significantly over time in both groups (P<0.001).

The LSD post hoc test showed that, in the intradermal group, the mean VAS score immediately after treatment was significantly lower than before treatment (P<0.001), and the score one month after treatment was lower than immediately after treatment (P=0.001). In the subcutaneous group, the mean VAS score immediately after treatment and one month after treatment was significantly lower than before treatment (P<0.001); however, the score one month after treatment was higher than immediately after treatment (P=0.01). Changes in VAS scores are shown in Figure-2. As shown in Table3, the independent-samples t-test showed that the mean dysfunction score before treatment did not differ significantly between the two groups (P=0.50). However, the mean dysfunction score was significantly lower in the intradermal mesotherapy group than in the subcutaneous mesotherapy group immediately after treatment (P=0.02) and one month after treatment (P<0.001). Repeated-measures ANOVA showed that mean dysfunction scores changed significantly over time in both groups (P<0.001). The LSD post hoc test showed that, in the intradermal group, the mean dysfunction score immediately after treatment and one month after treatment was significantly lower than before treatment (P<0.001), and the score one month after treatment was lower than immediately after treatment (P=0.005). In the subcutaneous group, the mean dysfunction score immediately after treatment and one month after treatment was significantly lower than before treatment (P<0.001), but there was no significant difference between immediately after treatment and one month after treatment (P=0.63). Changes in dysfunction scores are shown in Figure-3.

## Discussion

This randomized clinical trial compared the effectiveness of intradermal and subcutaneous mesotherapy for pain reduction and functional improvement in patients with KOA. Pain scores changed differently over time between groups. Pain initially decreased in the subcutaneous group but increased slightly by the one-month follow-up, whereas the intradermal group showed a sustained reduction in pain with further improvement over time. Although both methods improved function, intradermal mesotherapy resulted in greater and more sustained functional improvement over the four-week follow-up period. These time-dependent changes were accounted for in the repeated-measures analysis, which treated time as a within-subject factor.

The therapeutic effects of mesotherapy may extend beyond pharmacological action, as this technique can engage pain-modulating mechanisms within the skin. The dermis, including glial-cell-related mechanisms, may contribute to pain modulation [14]. This may partly explain the different analgesic effects observed at different injection depths.

Previous studies have supported the potential role of mesotherapy in musculoskeletal disorders. Faetani et al. reported that mesotherapy may alleviate pain, improve function, and facilitate early rehabilitation, thereby enhancing quality of life [15]. Because many patients experience pain relief after approximately five treatment sessions, clinicians should emphasize adherence to the complete treatment regimen and the role of rehabilitation in long-term recovery [16].

The effectiveness of mesotherapy may vary according to injection technique and target area. In a study of chronic low back pain, mesotherapy administered at acupuncture points produced greater short-term pain relief than trigger-point mesotherapy [17]. In the present study, six acupuncture points around the knee were selected as injection sites.

Mesotherapy has also demonstrated efficacy in acute pain conditions. In patients with lumbar disc herniation, intradermal mesotherapy produced greater pain reduction than intravenous dexketoprofen at multiple time points, including 24 h after treatment, and the systemic therapy group required analgesics more frequently [18].

Mesotherapy has also been evaluated for bilateral cervicobrachial pain. Ranieri et al. treated 10 patients with mesotherapy using diclofenac and lidocaine diluted in saline injected into the trapezius muscles with 0.23×12 mm needles and reported improvements in pain, functional status, myometric tone, and stiffness [19]. Scaturro et al. reported that intradermal mesotherapy with diclofenac and thiocolchicoside at a depth of 1-3 mm was effective and safe for treating cervical pain in patients with fibromyalgia [20]. In another clinical trial, both focal extracorporeal shock wave therapy and mesotherapy with thiocolchicoside and mepivacaine reduced myofascial symptoms, although extracorporeal shock wave therapy showed superior outcomes [21].

Chen et al. reported that mesotherapy using both profound and superficial intradermal injections was effective and safe for KOA at six-month follow-up. The mesotherapy group showed better outcomes and fewer adverse effects than the group treated with oral diclofenac, and patients receiving oral NSAIDs more frequently requested repeated treatment because of recurrent pain [22].

Saggini et al. compared intradermal mesotherapy using sodium diclofenac (25 mg/mL, 1 mL) three times weekly for three weeks with oral diclofenac (50 mg/day) in patients with Kellgren-Lawrence grade II KOA accompanied by pes anserine bursitis. Mesotherapy provided comparable pain relief with fewer adverse effects, and this benefit persisted for up to three months. Ultrasound evaluation also showed a reduction in the hypoechoic area associated with anserine bursitis only in the mesotherapy group [23].

A previous study showed that two sessions of subcutaneous mesotherapy at a 10-day interval using the same drug combination used in the present trial (20 mg/1 mL piroxicam and 2 mL of 2% lidocaine) were effective in patients with mild-to-moderate KOA. In that study, a 0.27×4 mm needle was inserted at a 30-45 degree angle into two to six painful points around the knee, suggesting that even a short course of subcutaneous mesotherapy may provide clinically meaningful pain relief in KOA [11].

Another randomized clinical trial compared mesotherapy injections containing an NSAID, local analgesic, vasodilator, cyanocobalamin, and physiological saline administered using point-by-point and nappage techniques with saline injections in patients with mild-to-moderate KOA. The mesotherapy group showed significantly greater pain relief and functional improvement than the saline group; at 16 weeks, the mean improvement in VAS score was 3.3 in the mesotherapy group compared with 0.2 in the saline group [24]. This study differed from the present trial in injection protocol and follow-up duration, and its longer follow-up provided additional information on the durability of mesotherapy effects.

Murat et al. compared deep intradermal mesotherapy (4 mm depth, point-by-point technique) with superficial intradermal mesotherapy (1-2 mm depth, nappage technique) for chronic nonspecific neck pain. Both techniques improved pain and functional status immediately after treatment, but deep intradermal injection produced greater short-term improvement; this difference was not significant at three months [25]. These findings differ from the present results, which showed superior pain reduction with intradermal injection at one-month follow-up. Differences in drug composition, injection technique, number of injection points, treatment frequency, and anatomical injection site may explain this discrepancy. Future studies could use ultrasonography to improve injection localization and optimize therapeutic efficacy.

The most common adverse event in the present study was transient burning pain at the injection site, which resolved within minutes. Ecchymosis was also observed and was more frequent in the intradermal group. No severe complications, including hypersensitivity reactions, infection, or dizziness, were recorded.

The main limitation of this study was the short follow-up duration. Patients were followed for only one month after completion of treatment; therefore, the long-term persistence of pain relief could not be assessed.

Future studies should evaluate long-term outcomes and establish standardized mesotherapy protocols, including optimal injection frequency, injection technique, and drug composition. Additional comparative trials are also needed to determine the relative effectiveness of mesotherapy compared with other available treatments.

## Conclusion

Mesotherapy was a well-tolerated treatment option for mild-to-moderate KOA and was associated with pain relief and functional improvement. Although both intradermal and subcutaneous mesotherapy were effective, intradermal mesotherapy resulted in superior pain reduction at one month after treatment and greater functional improvement compared with subcutaneous mesotherapy.

## Conflict of Interest

The authors declare that there are no conflicts of interest regarding the publication of this study.

## AI Disclosure Statement

During the preparation of this work, the authors used ChatGPT to check grammar, spelling, and the American style of writing for some sentences. After using this tool, the authors reviewed and edited the content as needed, taking full responsibility for the publication's content.
